# Usefulness of Mixed Reality in Surgical Treatment: Delphi Study

**DOI:** 10.2196/69964

**Published:** 2025-07-08

**Authors:** Renato Magalhães, Ana Carolina Lima, António Marques, Javier Pereira, Lúcio Lara Santos

**Affiliations:** 1 LabRP-CIR - Psychosocial Rehabilitation Laboratory - Center for Rehabilitation Research ESS, Polytechnic of Porto Porto Portugal; 2 ICBAS - Santo António Clinical Academic Center Porto Portugal; 3 Faculty of Engineering University of Porto Porto Portugal; 4 CITIC Research Center, Talionis Research Group Universidade da Coruña A Coruña Spain; 5 Experimental Pathology and Therapeutics Group Portuguese Oncology Institute of Porto (IPO Porto)/Porto Comprehensive Cancer Centre (Porto.CCC) & RISE@CI-IPOP (Health Research Network) Porto Portugal

**Keywords:** mixed reality, extended reality, augmented reality, operating rooms, surgery, consensus, Delphi study

## Abstract

**Background:**

Mixed reality (MR) combines real and virtual elements and has shown promise in diverse fields, including surgical procedures. MR headsets may support surgical navigation, planning, and training. It is crucial to determine whether medical professionals consider this technology indispensable. This study uses the Delphi method, facilitated by the Welphi web-based platform, to assess the utility of MR in surgical settings and analyzes the results of the first round using a systematic approach modeled on the PRISMA (Preferred Reporting Items for Systematic Reviews and Meta-Analyses) framework.

**Objective:**

This study aims to examine the feasibility and advantages of MR technology in surgical contexts. The findings are intended to inform and direct health care professionals, researchers, and developers in advancing MR integration into surgical environments to optimize treatment quality and safety.

**Methods:**

A 3-round Delphi approach was implemented to ascertain consensus on the utility of MR in surgical treatment. Participants (n=22) were purposefully selected from among experts with professional experience in technologies such as virtual reality, augmented reality, 3D laparoscopy, and robotics. In the first round, participants provided insights into the potential applications of MR in surgical procedures through open-ended questions structured across 5 distinct sections. Responses were analyzed to develop the second-round questionnaire, which was hierarchically organized into main topics and subtopics. In the third round, the questions were identical to those in the second round, including the percentage results, allowing participants to reconsider their responses. A consensus round was subsequently conducted. The majority consensus level was defined as agreement by ≥70% of the participants in a given round.

**Results:**

The study was conducted from January to May 2024. All 22 invited experts provided responses in both the first and second rounds (100% response rate). In the third and consensus rounds, 20 (91%) of the 22 experts participated. The consensus round, conducted to present the results, yielded a majority consensus (19/20, 95%) on the usefulness of MR in surgical treatment. The primary benefits of MR in surgery were identified as surgical navigation (15/20, 75%), planning (15/20, 75%), and teaching and training (14/20, 70%). In addition, 75% (15/20) of the experts identified cost and investments as primary constraints. We used the Kendall tau-b coefficient for correlation analysis, and significant correlations were identified between distinct aspects.

**Conclusions:**

MR technology is most beneficial in surgical navigation, planning, and training. However, the costs and investments required for implementation may present a potential limitation for the integration of this technology into surgical procedures. Moreover, it is of crucial importance to consider the ethical implications associated with MR use, particularly regarding patient safety and privacy.

## Introduction

### Background

Mixed reality (MR) is a technology that enables users to interact with and visualize both physical and virtual environments. It is increasingly being used in health care, with the global market projected to expand significantly over the next decade [1,2].

The global market for MR in health care was estimated at US $3 billion in 2023 and is projected to increase to US $3.58 billion in 2025 and US $7.86 billion in 2034 [2]. This increase, representing a compound annual growth rate of 9% from 2025 to 2034, reflects increasing investment and use of MR technologies in health care.

MR is distinct from augmented reality (AR), where the user can only see the virtual objects overlaid onto the physical world but cannot interact with them. It also differs from virtual reality (VR), where the user can visualize a virtual environment but is unable to perceive the real world [3,4].

There is considerable divergence among professionals in their understanding of the use of MR in surgical procedures. This discrepancy underscores the existence of significant gaps in comprehension and familiarity with this innovative technology. It is of the utmost importance to recognize and understand the diverse range of knowledge and expertise that exists among health professionals. This diversity of perspectives underscores the urgent need to identify and address specific gaps in the understanding regarding this technological tool. Conversely, to facilitate substantial advancements in this field, it is imperative to standardize the available information. The standardization of knowledge regarding the use of MR in surgical procedures can facilitate the establishment of a more consistent foundation for future investigations.

### Objectives

Accordingly, the objective of this Delphi study was 2-fold: first, to facilitate the homogenization of information and, second, to establish a robust foundation for the effective implementation of this innovative technology in the surgical environment. This will contribute to the continuous evolution of medical practice.

To inform this study, a thorough understanding of the topic of MR in surgical contexts was first achieved through a systematic review. This review enabled the identification as well as a comprehensive analysis, evaluation, and synthesis of all available evidence on the use of MR in the operating room [5]. The conclusions drawn from the reviewed articles highlight the promising contribution of these innovations to surgical practice, emphasizing notable benefits. Nevertheless, considerable obstacles were identified that must be surmounted for the more pervasive implementation of MR. The technical complexity, inflated costs, and steep learning curves associated with MR present substantial obstacles to its broader integration into the surgical setting. This highlights the necessity for the development of solutions that can facilitate its adoption in this field.

Numerous MR headsets are currently available, including the Microsoft HoloLens 2 [6], Magic Leap 2 [7], and Meta Quest Pro [8]. In particular, the Microsoft HoloLens 2 has been widely applied in health care, including through the VSI HoloMedicine [6,9]. A review of the literature revealed its use in various surgical contexts, including intraoperative use [10] as well as planning and training [9,11].

The operating room is a challenging environment for surgeons, with a number of difficulties being encountered. These include accurate documentation of surgical procedures, which may be addressed through the intraoperative capture of multimedia content [12]. Another challenge is the potential for human error, where surgical simulation [13] may be beneficial to enhance surgeons’ confidence [12]. In addition, real-time communication between operating rooms and specialists is crucial for delivering quality patient care [12].

Through the use of cutting-edge analytical techniques and a review of pertinent literature, we identified the main applications of MR in surgery: training [14-16], planning [17], simulation [18], and intraoperative applications such as surgical navigation [19]. Computed tomography data can be used to generate 3D models of the patient’s anatomical structures, which can then be viewed with an MR headset, such as the Microsoft HoloLens 2 [15,20]. MR also enables surgeons to communicate remotely and in real time [21], which is beneficial in situations where immediate communication is necessary. Furthermore, MR-based surgical simulations [18] and training enhance confidence among surgeons and trainees by allowing them to make and correct mistakes in a controlled environment.

MR also improves the safety and accuracy [22] of surgical procedures and may reduce x-ray exposure [10] in procedures that use a C-arm, a device used to obtain x-ray images during orthopedic surgery [23].

Of note, MR and AR technologies have certain limitations [24], which may be overcome through integration with other technologies. One potential limitation is eye fatigue [25], which can result from prolonged use. However, the use of 5G technology [26,27], which offers higher internet speeds and lower latency levels [28,29], is capable of mitigating this issue. Furthermore, some registration issues have been observed [30], whereby virtual objects fail to align with their physical counterparts [31]. This challenge could potentially be addressed by integrating MR with 3D printing technology [32,33].

There might be some issues regarding equipment sterility, namely with the Microsoft HoloLens 2. However, the implementation of voice commands removes the need for any physical interaction between the surgeon and the headset [34].

With regard to intraoperative applications, it would be beneficial to conduct a more comprehensive study on the regulations and ethical aspects involved [35], as well as to undertake additional projects with control groups [20]. This would facilitate a comparison of MR use with more traditional approaches. Moreover, a larger study population [20] would be typically required, as well as longitudinal data collection [10]. Although some studies have explored the cost-effectiveness [14,26] of MR, further research is required in this area. Furthermore, it would be beneficial to conduct a more in-depth investigation into the impact of eye fatigue and physical strain [36] when using these headsets, particularly when they are used for extended periods. In addition, it would be advantageous to assess the comfort level of the headsets in terms of weight [22] and design.

By analyzing past experiences, reviewing relevant research, and consulting experts in the field, we aim to understand the benefits of using MR in the operating room. The study seeks to provide valuable insights into MR’s effectiveness, acceptance, and potential challenges, as well as investigate how MR technology can enhance surgical procedures.

## Methods

### Design

To ascertain consensus on the utility of MR in surgical treatment, a Delphi study was used for the evaluation of this technology in surgical contexts. The majority consensus level was defined as agreement by ≥70% of the participants in a given round. This value was defined based on the literature, where the definition of consensus in Delphi studies can vary widely, with agreement percentages ranging from 50% to 97%, with 75% being the median value [37]. The Delphi method is a systematic technique for the collection and transformation of individual expert opinions into a group consensus through the administration of multiple rounds of surveys [38,39]. This methodology has been widely used in health-related research, including public health studies, the evaluation of health technologies, and the development of surgical education programs [40]. The Delphi method, by virtue of its anonymized application and iterative feedback regarding each expert’s opinions and contributions, serves to protect against the undue influence of any single expert in shaping the consensus. Furthermore, it reduces the likelihood of experts aligning with the group’s opinion, regardless of the evidence supporting their individual perspectives [41].

The Delphi method was used in this study to ensure a systematic approach to knowledge gathering, guarantee participant anonymity to mitigate individual influence, and protect against conformity bias. The Delphi process allows for a comprehensive assessment of perspectives from diverse surgical experts on the utility of MR in surgical procedures. Furthermore, this methodology minimizes the potential for bias, thereby promoting a robust and unbiased exploration of expert opinions.

We conducted this Delphi study on the web, managed through the Welphi platform [42], which is a platform designed specifically for Delphi studies.

### Participants

A diverse cohort of 22 nationally renowned specialists from a range of medical fields, including general, orthopedic, gynecological, pediatric, vascular surgery, anesthesiology, and interventional radiology, were invited to participate. At the time of the study, these experts, with an average of 18.18 (SD 5.26) years of professional experience in surgical treatment, were employed across various regions of Portugal. They also had an average of 7.27 (SD 5.16) years of experience with immersive technologies, such as MR, AR, VR, 3D laparoscopy, and robotics, with each participant having at least 1 year of such experience specifically applied in surgical settings.

All invited experts expressed interest in participating in the Delphi study. It is important to note that the surgical specialties listed were not the basis for inclusion in the sample; rather, the primary criterion for participation was being a surgeon working in Portugal with at least 1 year of experience using immersive technologies.

### Questionnaire Development and Implementation

**Figure 1:**
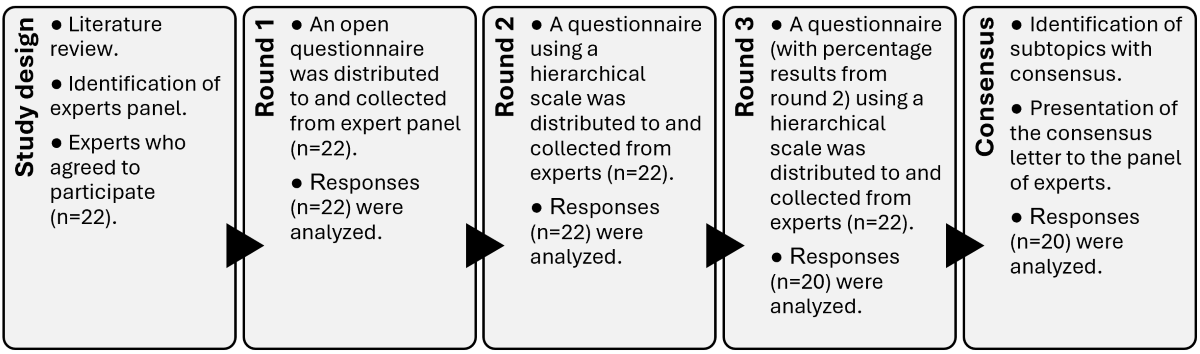
Overview of the Delphi study process.

A 3-round Delphi approach was conducted between January and May 2024 to achieve expert consensus on related questions regarding the usefulness of MR in surgical treatment. The first author was designated as the facilitator, tasked with identifying the expert panel and issuing formal invitations and reminders. The research team organized the questionnaires and oversaw the study’s implementation. The Delphi framework used for data collection and analysis is presented in Figure 1.

The first round of this Delphi study was structured into 5 sections with 13 questions. The first section gathered data about the experts invited to participate. The second section focused on their perspectives on MR in surgery, exploring current practices and potential applications. The third section assessed the perceived potential of MR to improve surgical procedures. Ethical and safety considerations, critical to the integration of MR into the surgical domain, were addressed in the fourth section. Finally, in the fifth section, the experts were invited to provide any additional comments. The first 4 sections had 3 questions each; the final section had only 1 question. The questionnaire used in the first round is presented in Multimedia Appendix 1.

In the second round, the questions were derived from the analysis of data from the first round. This round was organized into 5 thematic sections, and the experts were required to rank the provided statements in order of importance, from the most to the least important. The first section pertained to the utility of MR in surgical procedures, with 3 statements to be ranked. The second section addressed the applications of MR in surgery and included 4 statements. The third section, titled “Benefits of MR in surgery,” required the experts to rank 4 statements. The fourth section concerned the constraints associated with the implementation of MR in surgical settings and included 5 statements. The fifth and final section delved into ethical considerations and presented 4 statements for ranking.

In the third round, the same questions were posed as in the second round, but with 1 additional element: the experts were permitted to view the group’s responses from the second round, with the aim of potentially influencing their own subsequent responses and thereby reaching a consensus.

Finally, the experts were presented with the results in a consensus letter.

### Ethical Considerations

This study was conducted according to the ethical principles that have their origin in the Declaration of Helsinki and was approved by the Board Direction of the Surgery Department of IPO-Porto (December 19, 2023). All participants read the participant information sheet outlining the study objectives and procedures and gave informed consent before entering the study.

## Results

### Participants

A total of 22 experts were invited to participate in this Delphi study, of whom 20 (91%) completed the third round. In most of the sections, a majority consensus (≥70%) was reached on at least 1 item. The experts’ responses in the third round were analyzed using the Kendall tau-b correlation to evaluate the degree of concordance. Significant correlations were identified across various sections, indicating the strength and direction of associations between variables. Key findings included that 75% (15/20) of the experts indicated that the primary utility of MR was surgical navigation, and 80% (16/20) agreed that the predominant application of MR in surgery was surgical orientation and navigation. In addition, 75% (15/20) indicated that accuracy and results improvement were the most significant benefits of MR in surgery, while 75% (15/20) identified costs and investments as the primary constraints on the implementation of MR in surgical settings. Ethical considerations were also highlighted, with 70% (14/20) identifying the safety of procedures and patient protection as the most important concerns. In the final phase of the study, the findings and conclusions were presented to 20 (91%) of the 22 participants, who were then asked whether they wished to endorse them. An acceptance rate of 95% (19/20) was obtained, indicating strong agreement with the study’s conclusions.

### Round 1

The first-round results are presented according to the thematic areas addressed in each question. These results reflect substantial consensus among the experts. To analyze the data, a PRISMA (Preferred Reporting Items for Systematic Reviews and Meta-Analyses)-like approach was adopted [43]. As all questions were open ended, 2 investigators categorized the responses into areas of interest (ie, applications). These categorizations were then compared with each other by a third investigator, resulting in a consensus. The results for each question, along with the corresponding areas of interest, are summarized in the following tables. It should be noted that the number of responses displayed in each table correlates with the total number of responses assigned to a given area, considering the possibility of a response being included in multiple areas. An additional column in each table represents the number of participants who provided responses falling into each area of interest.

With regard to the first question, pertaining to the applications of MR in surgical procedures, 6 areas of interest were identified by the investigators and are presented in Table 1.

The second question pertained to the benefits of MR in surgical procedures. The experts’ responses were classified into 4 distinct categories, as shown in Table 2.

**Table 1:** Applications of mixed reality in medicine, especially in surgery (n=22).

Applications	Responses, n	Participants, n (%)
Surgical planning	12	10 (45)
Teaching and surgical training	14	13 (59)
Surgical orientation and navigation	17	15 (68)
Data integration and visualization	13	11 (50)
Safety and improvement of results	12	9 (41)
Communication and collaboration	7	7 (32)

**Table 2:** Main benefits of mixed reality in surgical procedures (n=22).

Main benefits	Responses, n	Participants, n (%)
Accuracy and improvement of results	24	15 (68)
Communication and collaboration	9	7 (32)
Efficiency and optimization	13	10 (45)
Safety	13	13 (59)

The potential limitations or challenges in relation to implementing MR in surgical procedures were similarly discussed and classified into 6 areas of interest, as shown in Table 3.

All experts agreed that MR has the potential to improve the precision and effectiveness of surgical procedures. Their responses were classified into 2 categories, as seen in Table 4.

The experts were asked whether they were aware of any applications of MR in surgical procedures. Of the 22 experts, 16 (73%) reported familiarity with at least 1 case. Of these 16 respondents, 2 (12%) were aware of 1 case, 8 (50%) were aware of 2 cases, and 6 (38%) were aware of 3 cases (Table 5).

The experts identified 8 surgical areas that may derive the greatest benefit from the application of MR technology (Table 6).

Six areas of interest were identified with regard to ethical concerns, as shown in Table 7.

**Table 3:** Limitations or challenges in relation to the implementation of mixed reality in surgical procedures (n=22).

Limitations or challenges	Responses, n	Participants, n (%)
Costs and investments	12	12 (55)
Technology related	19	14 (64)
Human challenges and technology adoption challenges	23	14 (64)
Logistics and operational	3	3 (14)
Certification	4	4 (18)
Other	1	1 (5)

**Table 4:** Potential of mixed reality to improve the precision and effectiveness of surgical procedures (n=22).

Categories	Responses, n	Participants, n (%)
Accuracy and results improvement	15	15 (68)
Efficiency and optimization	10	10 (45)

**Table 5:** Specific examples or use cases where mixed reality is already being successfully applied in surgery (n=22).

Specific examples or use cases	Responses, n	Participants, n (%)
Surgical planning	17	11 (50)
Visualization, real-time orientation, and intraoperative navigation	11	9 (41)
Education and surgical training	3	3 (14)
Robotic surgery	4	4 (18)
Other	4	4 (18)

**Table 6:** Specific surgical areas that might benefit the most from mixed reality technology (n=22).

Surgical areas	Responses, n	Participants, n (%)
General surgery	17	14 (64)
Neurosurgery	9	9 (41)
Orthopedics	12	12 (55)
Urology	7	6 (27)
Vascular surgery	9	9 (41)
Thoracic surgery	7	5 (23)
Interventional radiology	7	6 (27)
Other	2	2 (9)

**Table 7:** Ethical concerns to consider in the use of mixed reality in surgical procedures (n=22).

Ethical concerns	Responses, n	Participants, n (%)
Safety of procedures and patient protection	8	8 (36)
Responsibility and regulation	13	10 (45)
Privacy and patient confidentiality	15	14 (64)
Patient informed consent	5	5 (23)
Cost-benefit relationship	5	4 (18)
Other	3	3 (14)

Regarding patient privacy, of the 22 experts, 11 (50%) indicated that it might be compromised (eg, data privacy), while the remaining 11 (50%) believed that no issues existed in this regard.

Concerning patient safety, 20 (91%) of the 22 experts believed that it could be affected. Among these experts, opinions were divided, with some (3/22, 14%) indicating that the changes could be beneficial and others (17/22, 77%) suggesting the possibility of adverse effects. Only 2 (9%) of the 22 experts indicated that patient safety would remain unaffected.

The final question was not subjected to analysis because the supplementary remarks had already been addressed in the initial phase.

### Round 2

After the analysis of the responses provided by the experts in the first round, the initial questionnaire was adjusted based on the results, and a closed-ended questionnaire was developed for the second round. The primary topics examined with regard to the potential applications of MR in surgical treatment were utility, the areas of potential application, benefits, limitations, and ethical considerations. The objective of this round was to assign a ranking (1, 2, 3, and so on) to each item based on the data obtained from the previous round. Items deemed the most relevant were to be ranked highest, while those deemed the least relevant were to be ranked lowest.

The experts’ rankings of the statements are shown in Textbox 1.

**Experts’ rankings of the statements.**

**Utility of MR in surgical contexts**

1. Surgical navigation is the main usefulness of MR in surgery.
2. Surgical planning is the main usefulness of MR in surgery.
3. Surgical teaching and training is the main usefulness of MR in surgery.

**Section related to the applications of MR in surgery**

1. The main application of MR in surgery is surgical orientation and navigation.
2. The main application of MR in surgery is education and surgical training.
3. The main application of MR in surgery is surgical planning.
4. The main application of MR in surgery is integrating it with other surgical systems.

**Main benefits of MR in surgery**

1. The main benefit of MR in surgery is accuracy and results improvement.
2. The main benefit of MR in surgery is safety.
3. The main benefit of MR in surgery is efficiency and optimization.
4. The main benefit of MR in surgery is communication and collaboration.

**Limitations in the implementation of MR in surgery**

1. The main limitations in the implementation of MR in surgery are technology related.
2. The main limitations in the implementation of MR in surgery are people related.
3. The main limitations in the implementation of MR in surgery are the costs and investments.
4. The main limitation in the implementation of MR in surgery is the regulatory area.
5. The main limitation in the implementation of MR in surgery is data protection.

**Ethical considerations of MR in surgery**

1. The main ethical considerations of MR in surgery are patient privacy and confidentiality.
2. The main ethical considerations of MR in surgery are responsibility and regulation.
3. The main ethical considerations of MR in surgery are the safety of procedures and patient protection.
4. The main ethical consideration of MR in surgery is patient informed consent.

### Round 3

In this round, the same questions as in the previous round were presented, accompanied by visual representations showing the percentage of responses from the 22 participants, in accordance with the Delphi methodology. This allowed each participant to review their position in light of the group’s responses, thereby reducing individual bias and facilitating a collective understanding on the topic.

Of the 22 experts, 20 (91%) responded to this round. The results of the second and third rounds are presented in Tables 8-12. A majority consensus was reached in all sections, with a consensus level of ≥70%, meaning that at least 14 (70%) of the 20 participants agreed on each item. In all sections, the order of the answers remained consistent with the second round; however, a greater proportion of experts agreed with the top-ranked statement in each section.

**Table 8:** Results from the second and third rounds, grouped by the topic “utility of mixed reality (MR) in surgery.”

Rounds and ranking of items		Surgical navigation is the main usefulness of MR in surgery, n (%)	Surgical planning is the main usefulness of MR in surgery, n (%)	Surgical teaching and training is the main usefulness of MR in surgery, n (%)
**Round 2: item ranking (n=22)**				
	1	14 (64)	5 (23)	3 (14)
	2	4 (18)	12 (55)	6 (27)
	3	4 (18)	5 (23)	13 (59)
**Round 3: item ranking (n=20)**				
	1	15 (75)	2 (10)	2 (10)
	2	1 (5)	15 (75)	4 (20)
	3	4 (20)	3 (15)	14 (70)

**Table 9:** Results from the second and third rounds, grouped by the topic “areas of wider application of mixed reality (MR).”

Rounds and ranking of items		Surgical orientation and navigation, n (%)	Surgical education and training, n (%)	Surgical planning, n (%)	Integration of MR with other surgical systems (eg, robotics), n (%)
**Round 2: item ranking (n=22)**					
	1	13 (59)	3 (14)	5 (23)	1 (5)
	2	4 (18)	3 (14)	8 (36)	7 (32)
	3	3 (14)	7 (32)	4 (18)	9 (41)
	4	2 (9)	9 (41)	5 (23)	5 (23)
**Round 3: item ranking (n=20)**					
	1	16 (80)	2 (10)	2 (10)	0 (0)
	2	2 (10)	3 (15)	10 (50)	5 (25)
	3	1 (5)	8 (40)	2 (10)	11 (55)
	4	1 (5)	7 (35)	6 (30)	4 (20)

**Table 10:** Results from the second and third rounds, grouped by the topic “benefits of mixed reality in the surgical field.”

Rounds and ranking of items		Accuracy and improvement of results, n (%)	Security, n (%)	Efficiency and optimization, n (%)	Communication and collaboration, n (%)
**Round 2: item ranking (n=22)**					
	1	14 (64)	4 (18)	3 (14)	1 (5)
	2	5 (23)	11 (50)	5 (23)	1 (5)
	3	2 (9)	5 (23)	8 (36)	8 (36)
	4	1 (5)	2 (9)	6 (27)	12 (55)
**Round 3: item ranking (n=20)**					
	1	15 (75)	3 (15)	2 (10)	0 (0)
	2	3 (15)	14 (70)	2 (10)	1 (5)
	3	2 (10)	1 (5)	11 (55)	7 (35)
	4	0 (0)	2 (10)	5 (25)	12 (60)

**Table 11:** Results from the second and third rounds, grouped by the topic “limitations in the implementation of mixed reality in the surgical environment.”

Rounds and ranking of items		Technology related, n (%)	Human challenges and technology adoption, n (%)	Costs and investments, n (%)	Regulatory area, n (%)	Data protection, n (%)
**Round 2: item ranking (n=22)**						
	1	5 (23)	1 (5)	14 (64)	2 (9)	0 (0)
	2	8 (36)	7 (32)	1 (5)	5 (23)	1 (5)
	3	3 (14)	9 (41)	3 (14)	4 (18)	4 (18)
	4	3 (14)	3 (14)	2 (9)	10 (45)	4 (18)
	5	3 (14)	2 (9)	2 (9)	1 (5)	13 (59)
**Round 3: item ranking (n=20)**						
	1	4 (20)	0 (0)	15 (75)	1 (5)	0 (0)
	2	10 (50)	5 (25)	2 (10)	3 (15)	0 (0)
	3	3 (15)	13 (65)	2 (10)	1 (5)	2 (10)
	4	2 (10)	2 (10)	0 (0)	14 (70)	3 (15)
	5	1 (5)	0 (0)	1 (5)	1 (5)	15 (75)

**Table 12:** Results from the second and third rounds, grouped by the topic “ethical considerations in the use of mixed reality in the surgical environment.”

Rounds and ranking of items		Patient privacy and confidentiality, n (%)	Responsibility and regulation, n (%)	Safety of procedures and patient protection, n (%)	Patient informed consent, n (%)
**Round 2: item ranking (n=22)**					
	1	1 (5)	6 (27)	12 (55)	3 (14)
	2	5 (23)	10 (45)	4 (18)	3 (14)
	3	8 (36)	4 (18)	3 (14)	7 (32)
	4	8 (36)	2 (9)	3 (14)	9 (41)
**Round 3: item ranking (n=20)**					
	1	1 (5)	4 (20)	14 (70)	2 (10)
	2	4 (20)	11 (55)	3 (15)	2 (10)
	3	8 (40)	4 (20)	1 (5)	7 (35)
	4	7 (35)	1 (5)	2 (10)	9 (45)

To facilitate the interpretation of the results, the Kendall tau-b correlation was chosen for this study due to its effectiveness in analyzing associations between ordinal variables. This test is particularly advantageous for evaluating the concordance between rankings because it accurately accounts for tied ranks. Kendall tau-b provides a precise measure of the association between variables, making it an ideal tool for ordinal data analysis. All results, with the coefficients indicating the strength and direction of the association between 2 ordinal variables, are presented in Multimedia Appendix 2. A negative value suggests that, in general, when one variable is rated higher, the other tends to be rated lower, and vice versa. Statistical significance was defined as *P*<.05. All calculations were performed using SPSS software (version 29.0; IBM Corp).

In the first section, 15 (75%) of the 20 experts indicated that the primary utility of MR was surgical navigation. In addition, 16 (80%) of the 20 experts agreed that the predominant application of MR in surgery was surgical orientation and navigation. For “surgical teaching and training,” the mode was 3; and for “surgical planning,” it was 2. The Kendall tau-b correlation between these 2 variables was weak, negative, and not significant (Kendall tau-b=−0.313; *P*=.15). This implied that there was insufficient evidence to conclude that a relationship existed between “surgical teaching and training” and “surgical planning” as perceived by the participants in question. Consequently, it was not feasible to ascertain a meaningful relationship between these 2 variables, based on the available data.

For “surgical teaching and training,” the mode was 3; and for “surgical navigation,” it was 1. The Kendall tau-b correlation between these 2 variables was moderate, negative, and significant (Kendall tau-b=−0.457; *P*=.04). As the experts rated “surgical teaching and training” higher, they tended to rate “surgical navigation” lower, and vice versa.

For “surgical planning,” the mode was 2; and for “surgical navigation,” it was 1. The Kendall tau-b correlation between these 2 variables was moderate, negative, and significant (Kendall tau-b=−0.450; *P*=.04). This indicates that participants who rated “surgical planning” higher tended to rate “surgical navigation” lower, and vice versa (Table S1 in Multimedia Appendix 2).

In the section on the applications of MR in surgery, significant Kendall tau-b correlations were found. Between “integrating it with other surgical systems” and “surgical planning,” there was a moderate, negative, and significant correlation (Kendall tau-b=−0.567; *P*=.006). In addition, “surgical planning” and “surgical orientation and navigation” showed a moderate, negative, and significant correlation (Kendall tau-b=−0.468; *P*=.03). For “integrating it with other surgical systems,” the mode was 3; for “surgical planning,” it was 2; and for “surgical orientation and navigation,” it was 1. Thus, it was concluded that the experts who rated “integrating it with other surgical systems” higher tended to rate “surgical planning” lower, and vice versa. Similarly, the experts who rated “surgical planning” higher tended to rate “surgical orientation and navigation” lower, and vice versa (Table S2 in Multimedia Appendix 2).

In the section “benefits of MR in surgery,” 15 (75%) of the 20 experts indicated that accuracy and results improvement were the most significant benefits of MR in surgery. Significant Kendall tau-b correlations were identified. Between “communication and collaboration” and “efficiency and optimization,” there was a moderate, negative, and significant correlation (Kendall tau-b=−0.435; *P*=.04). In addition, “efficiency and optimization” and “safety” showed a strong, negative, and significant correlation (Kendall tau-b=−0.740; *P*<.001). For “communication and collaboration,” the mode was 4; for “efficiency and optimization,” it was 3; and for “safety,” it was 2. These results indicate a correlation between the ratings of “communication and collaboration” and “efficiency and optimization,” with higher ratings of the former tending to correspond with lower ratings of the latter, and vice versa (Table S3 in Multimedia Appendix 2).

A majority of the experts (15/20, 75%) identified costs and investments as the primary constraints on the implementation of MR in surgical settings. Significant Kendall tau-b correlations were identified. Between “data protection” and “costs and investments,” there was a moderate, negative, and significant correlation (Kendall tau-b=−0.476; *P*=.03). For “data protection,” the mode was 5; and for “costs and investments,” it was 1. Similarly, a moderate, negative, and significant correlation was observed between “regulatory area” and “technology related” (Kendall tau-b=−0.457; *P*=.02). For “regulatory area,” the mode was 4; and for “technology related,” it was 2. In addition, a moderate, negative, and significant correlation was noted between “costs and investments” and “technology related” (Kendall tau-b=−0.416; *P*=.04). These results indicate a correlation between the ratings of “data protection” and “costs and investments,” with higher ratings for the former tending to correspond with lower ratings for the latter (Table S4 in Multimedia Appendix 2).

The most important ethical considerations, as identified by 14 (70%) of the 20 experts, pertain to the safety of procedures and patient protection. It was possible to identify significant Kendall tau-b correlations between “safety of procedures and patient protection” and “responsibility and regulation.” These correlations were moderate, negative, and significant (Kendall tau-b=−0.416; *P*=.04), indicating that participants who rated “safety of procedures and patient protection” higher tended to rate “responsibility and regulation” lower, and vice versa (Table S5 in Multimedia Appendix 2). For “safety of procedures and patient protection,” the mode was 1; and for “responsibility and regulation,” it was 2.

Table 13 identifies the primary topics and subtopics with a majority consensus of ≥70%, meaning that at least 14 (70%) of the 20 experts agreed on each point, including their ranking (1, 2, 3, 4, and 5), as determined by the panel.

**Table 13:** Main topics and subtopics with a majority consensus of ≥70%, including ranking (n=20).

Main topics and subtopics			Ranking		Participants agreeing, n (%)
**Utility of MR^a^**					
	Surgical navigation is the main usefulness of MR in surgery	1		15 (75)	
	Surgical planning is the main usefulness of MR in surgery	2		15 (75)	
	Surgical teaching and training is the main usefulness of MR in surgery	3		14 (70)	
**Areas of wider application of MR**					
	Surgical orientation and navigation	1		16 (80)	
**Benefits of MR in the surgical field**					
	Accuracy and improvement of results	1		15 (75)	
	Security	2		14 (70)	
**Limitations in the implementation of MR in the surgical environment**					
	Costs and investments	1		15 (75)	
	Regulatory area	4		14 (70)	
	Data protection	5		15 (75)	
**Ethical considerations in the use of MR in the surgical environment**					
	Safety of procedures and patient protection	1		14 (70)	

^a^MR: mixed reality.

### Consensus Round

In this phase of the study, the findings and conclusions were presented to 20 experts, who were then asked whether they wished to endorse them. An acceptance rate of 95% (19/20) was obtained.

### Principal Results

#### Usefulness of MR in Surgical Treatment

The aspects of MR’s usefulness in surgery, mentioned subsequently, were identified through expert consensus.

“Surgical navigation is the main usefulness of MR in surgery” obtained a majority consensus of 75% (15/20), meaning that surgical navigation was ranked first in terms of MR’s usefulness in surgical treatment. Using MR for surgical navigation allows the surgeon to improve accuracy when identifying critical anatomical structures and aids in decision-making [44].

“Surgical planning is the main usefulness of MR in surgery” obtained a majority consensus of 75% (15/20), meaning that surgical planning was ranked second in terms of MR’s usefulness in surgical treatment. Using MR for surgical planning allows the surgeon to visualize procedures in detail before surgery, simulate various surgical approaches, define the most appropriate action plan, and anticipate potential complications and challenges [17].

“Surgical teaching and training is the main usefulness of MR in surgery” obtained a majority consensus of 70% (14/20), meaning that surgical teaching and training was ranked third in terms of MR’s usefulness in surgical treatment. Using MR for surgical teaching and training allows the practice of procedures in a holographic environment without risk to patients; offers real-time error feedback; and provides access to teaching and training from any location, thereby reducing dependence on university simulators [11].

#### MR Application Areas

The findings regarding the practical applications of MR in surgical treatment are mentioned subsequently.

“Surgical orientation and navigation” obtained a majority consensus of 80% (16/20), ranking it as the leading application area for MR in surgical treatment. Using MR for surgical orientation and navigation assists the surgeon in real time to perform cuts and interventions with greater precision, minimizing damage to healthy tissues [19].

The other subtopics—“surgical planning,” “integration of MR with other surgical systems (eg, robotics),” and “education and surgical training”—did not achieve a significant majority consensus.

#### Benefits of MR in the Surgical Field

The findings regarding the benefits of MR in the surgical field are mentioned subsequently.

“Accuracy and improvement of results” was agreed upon by 75% (15/20) of the experts, ranking it as the first benefit of MR in the surgical field. Using MR benefits the surgeon by allowing the visualization of 3D holographic images superimposed on the field of surgical intervention, allowing for better precision, which consequently may lead to a reduction in postoperative complications, such as infections, bleeding, and accidental injuries [34].

“Security” was agreed upon by 70% (14/20) of the experts, ranking it as the second benefit of MR in the surgical field. MR applied to surgery allows for continuous and accurate visual assistance, assisting surgeons in decision-making and providing visual alerts when approaching critical areas [22].

The other subtopics—“efficiency and optimization” and “communication and collaboration”—did not achieve a significant majority consensus.

#### Limitations in the Implementation of MR in the Surgical Environment

The findings regarding limitations in the implementation of MR in the surgical environment are mentioned subsequently.

“Costs and investments” was agreed upon by 75% (15/20) of the experts, ranking this topic as the leading challenge to the implementation of MR in surgery. The initial investment involves the acquisition of advanced headsets and specialized software, integration with clinical systems, and the installation of up-to-date wired and wireless network infrastructures [45].

“Data protection” also obtained a majority consensus of 75% (15/20), it was ranked fifth based on experts’ prioritization. This indicates that systems must ensure the protection of sensitive patient data, complying with the European Union’s General Data Protection Regulation [35].

“Regulatory area” obtained a majority consensus of 70% (14/20), ranking it as fourth in terms of limitations in the implementation of MR in surgery, considering that the approval of new MR devices and software by regulators is a lengthy and complex process [46].

The other subtopics—“human challenges” and “technology adoption challenges”—did not reach a significant majority consensus, with divergence in the rankings. These points can be explored in future research or discussions.

#### Ethical Considerations in the Use of MR in the Surgical Field

The findings regarding ethical considerations in the use of MR in the surgical field are mentioned subsequently.

“Safety of procedures and patient protection” was agreed upon by 70% (14/20) of the experts, ranking it as the leading ethical consideration in the implementation of MR in surgery. It is crucial to ensure that MR devices are highly reliable and that they function correctly during surgical procedures because any technical failure can compromise patient safety [47].

There was no majority consensus on the ranking of the subtopics “patient privacy and confidentiality,” “responsibility and regulation,” and “patient informed consent.” However, there was a tendency to rank “responsibility and regulation” second and “patient informed consent” last.

## Discussion

### Principal Findings

The principal findings of this study suggest that MR technology possesses substantial potential to enhance surgical procedures by improving orientation and navigation, increasing accuracy and outcomes, and facilitating real-time communication. Unlike VR or AR, MR enables unique, real-time interaction with holographic objects within the spatial context. This allows for seamless integration with existing surgical technologies, suggesting an already identified potential. Despite the potential financial implications of implementing MR in surgery, such as costs related to software development, professional training, or hardware, there was consensus among the experts that the use of MR in surgical settings is justified. Moreover, MR equipment (hardware) used in surgical contexts has been described in the literature as “low cost” [14]*.* At the level of implementation science, the adoption of MR in surgical contexts, as with other technologies such as robotics, requires appropriate training programs and standardized protocols. Ethical considerations are primarily centered on the safety of procedures and patient protection.

The primary utility of MR in surgical settings was initially perceived to be its capacity to support surgical orientation and navigation, which was identified as the predominant application. MR has been used for this purpose extensively [19]. The experts hypothesized that the accuracy and outcomes of surgical procedures may be enhanced through the use of MR. This is supported by the findings of Fidan et al [19], who demonstrated that MR tools assisted in the decision-making process during surgery by displaying 3D holograms of the patient’s anatomical structures. However, a potential obstacle to the adoption of this technology is the cost of the equipment.

In this study, the primary ethical concerns pertained to the safety of procedures and patient protection. This is consistent with the findings of Lam et al [47], who identified regulatory aspects and patient welfare as the most significant ethical considerations. Patient autonomy is a fundamental ethical principle that must be respected in MR-assisted surgeries. Any surgical intervention must be preceded by the patient’s informed consent, which should include information about the benefits of the technology, risks, and alternatives, in accordance with the patient’s rights and informed decision-making [48]. It is important to define clear protocols to delineate the roles and coordination among individuals involved in procedures using 3D model visualization. This includes outlining responsibilities for the person directly managing the 3D visualization, as well as for the rest of the surgical team, nurses, and technicians. This is essential for maintaining standards of care and for assessing and intervening in any complications that may arise [49]. These protocols should include the definition of who is responsible for manipulating the MR devices, monitoring clinical data, maintaining communication, and providing technical support during the procedure [50].

All initial statements were endorsed by the majority (19/20, 95%) of the experts in this Delphi study. The experts concurred that the use of MR in surgery is justified, despite the potential financial considerations associated with the necessary equipment. The opportunity to view group responses after the second round enabled the experts to reconsider their initial rankings, thereby facilitating the attainment of consensus. To minimize the potential influence of authority figures [41,47], all rounds of the study were conducted anonymously.

### Comparison With Prior Work

Although this is an emerging topic with limited published research, particularly Delphi surveys related to MR and extended reality in surgical contexts, some studies present similar objectives.

In Lam et al [47], for instance, the objective of the 4-round Delphi study was to establish a definition for the term “digital surgery.” This was an international Delphi study involving 38 experts, compared to the 22 experts included in our study. The consensus level of 70% was identical to that observed in our study. However, in Lam et al [47], the experts participated in a web-based meeting, which was not part of our study design. The main takeaways from the study [47] that could be related to those from our study concerned ethical issues that developers need to consider when creating digital applications for surgical use [47].

In Burke et al [51], 43 surgeons participated in a Delphi study. Similar to our study, it comprised 3 rounds, with some questions in the second and third rounds developed based on responses from previous rounds. The level of consensus was slightly higher, at 80%. The goal of this survey [51] was to provide educational stakeholders with a comprehensive overview of a robotic surgery curriculum from the perspective of medical students.

Finally, in Burian et al [52], the Delphi study focused on astronaut medical training using extended reality, which is comparable to one of the applications mentioned in our study: MR for surgical training. The study included 45 participants and used a modified Delphi method, incorporating web-based meetings. A 7-point Likert scale was used, which might have been beneficial in our study as well, potentially offering additional information on the topic. The main conclusions of the study by Burian et al [52] pertained to the extended reality functionalities required for medical training and real-time clinical support during deep space missions. Similarly, our study offered insights into the needs of surgeons in the operating room, such as the use of MR for surgical navigation and training.

### Limitations

The conclusions derived from this Delphi exercise reflect the subjective opinions of a specific group of experts, albeit one composed of specialists from various surgical fields.

Future research could consider repeating this study using a Likert scale to verify whether the same conclusions are reached. A larger number of experts in the sample is recommended to achieve stronger results. This study was conducted exclusively within the Portuguese context; it would be of interest to assess the impact of MR tools with an international panel. Regarding the results, it is notable that certain areas exhibited lower levels of consensus than others; for instance, ethical considerations reached agreement among only 14 (70%) of the 20 experts. Further investigation into the discrepancies between surgical specialties may prove beneficial.

Cost-effectiveness and a cost-benefit analysis were not included in this study; however, such evaluations could be useful in future research to support managerial decision-making regarding investment in these technologies. Future investigations should also address economic aspects, including the return on investment and the costs associated with maintenance and system updates. In our study, additional investigation into potential technical barriers may have been beneficial. In future studies, it is recommended to consider the technical specifications required for the implementation of these tools.

In addition, it would be beneficial to evaluate the accuracy and complication rates associated with MR-assisted procedures. It would also be advantageous to identify the learning curve associated with each type of surgical procedure.

### Conclusions

The results of this Delphi study provide a comprehensive overview of experts’ perceptions regarding the usefulness of MR in surgery. Surgical navigation, planning, and teaching and training were identified as the areas of greatest utility, while improved accuracy and enhanced safety emerged as the main benefits. However, excessive costs and regulatory barriers were cited as potential limitations to implementation. Ethical considerations, especially in relation to patient safety, are crucial to fostering trust and acceptance of MR in surgical practice.

As a next step, we intend to develop a tool for surgical teaching and training using MR, which will allow us to obtain data on surgical navigation, planning, and process-related limitations. Subsequently, a proof-of-concept study will be conducted in a selected application area, through which metrics will be developed to evaluate feasibility, accuracy, and usefulness.

## Appendix

Multimedia Appendix 1 The questionnaire used in the first round of the Delphi study.

Multimedia Appendix 2 SPSS output tables showing Kendall tau-b correlations for key topics on the use of mixed reality in surgical treatment.

## Abbreviations

AR: augmented reality
MR: mixed reality
PRISMA: Preferred Reporting Items for Systematic Reviews and Meta-Analyses
VR: virtual reality
