# Supplementary material for: Usefulness of Mixed Reality in Surgical Treatment: Delphi Study
Source: J Med Internet Res. 2025 Jul 8;27:e69964. doi: 10.2196/69964 (PMC12284448; doi:10.2196/69964)
Supplement: Multimedia Appendix 1 [file jmir_v27i1e69964_app1.docx]

## Multimedia Appendix 1: The questions from the first round of the Delphi study

The questions from the first round of the Delphi study, as well as the areas of interest of each section, were presented as follows:

**Section 1: Participant information**

1. Position or title in the surgical field;
2. Years of professional experience in surgical treatment;
3. Years of professional experience in immersive or interactive technologies, such as virtual reality (VR), augmented reality (AR), 3D laparoscopy, robotics, or other similar technologies applied to surgery;

**Section 2: MR and surgery**

1. What, in your opinion, are the applications of Mixed Reality (MR) in medicine, especially in surgery? (Please identify up to three)
2. What are the main benefits that MR can offer to surgical procedures? (Please identify up to three)
3. What limitations or challenges do you identify in relation to the implementation of MR in surgical procedures? (Please identify up to three)

**Section 3: Potential of MR in the improvement of surgical procedures**

1. Do you believe that MR has the potential to improve the precision and effectiveness of surgical procedures? Yes/No. Please explain your answer.
2. Are you aware of specific examples or use cases where MR is already being successfully applied in surgery? Yes/No. If Yes, identify up to three.
3. What specific surgical areas might benefit the most from MR technology? (Please identify up to three)

**Section 4: Ethical and safety concerns**

1. What ethical concerns should be considered in the usage of MR in surgical procedures? (Please identify up to three)
2. Do you consider that patient privacy could be affected by the use of immersive technologies, such as VR, AR or other similar technologies, in surgery? Yes/No. Please explain your answer.
3. Do you consider that there may be changes to patient safety during the procedure when incorporating MR into surgical procedures? Yes/No. Please explain your answer.

**Section 5: Additional comments**

1. Which other aspects should be discussed?
