# Supplementary material for: Usefulness of Mixed Reality in Surgical Treatment: Delphi Study
Source: J Med Internet Res. 2025 Jul 8;27:e69964. doi: 10.2196/69964 (PMC12284448; doi:10.2196/69964)
Supplement: Multimedia Appendix 2 [file jmir_v27i1e69964_app2.docx]

## Multimedia Appendix 2: SPSS output tables showing Kendall tau-b correlations for key topics on the use of mixed reality in surgical treatment.

Table S1. Correlations of the 3 topic items of utility of Mixed Reality.

|  | | | SUR_TRAIN | SUR_PLAN | SUR_NAV |
| --- | --- | --- | --- | --- | --- |
| Kendall's tau_b | SUR_TRAIN | Correlation Coefficient | -- |  |  |
|  |  | Sig. (2-tailed) | . |  |  |
|  |  | N | 20 |  |  |
|  | SUR_PLAN | Correlation Coefficient | -,313 | -- |  |
|  |  | Sig. (2-tailed) | ,148 | . |  |
|  |  | N | 20 | 20 |  |
|  | SUR_NAV | Correlation Coefficient | -,457^a^ | -,450^a^ | -- |
|  |  | Sig. (2-tailed) | ,036 | ,040 | . |
|  |  | N | 20 | 20 | 20 |
| ^a^Correlation is significant at the 0.05 level (2-tailed). | | | | | |

Table S2. Correlations of the 4 topic items of Areas of Wider Application of Mixed Reality.

|  | | | INTEGRATION | PLANNING | EDUCATION | ORIENTATION |
| --- | --- | --- | --- | --- | --- | --- |
| Kendall's tau_b | INTEGRATION | Correlation Coefficient | -- |  |  |  |
|  |  | Sig. (2-tailed) | . |  |  |  |
|  |  | N | 20 |  |  |  |
|  | PLANNING | Correlation Coefficient | -,567^b^ | -- |  |  |
|  |  | Sig. (2-tailed) | ,006 | . |  |  |
|  |  | N | 20 | 20 |  |  |
|  | EDUCATION | Correlation Coefficient | -,258 | -,264 | -- |  |
|  |  | Sig. (2-tailed) | ,204 | ,187 | . |  |
|  |  | N | 20 | 20 | 20 |  |
|  | ORIENTATION | Correlation Coefficient | ,364 | -,468^a^ | -,298 | -- |
|  |  | Sig. (2-tailed) | ,086 | ,025 | ,150 | . |
|  |  | N | 20 | 20 | 20 | 20 |
| ^a^Correlation is significant at the 0.05 level (2-tailed). ^b^Correlation is significant at the 0.01 level (2-tailed). | | | | | | |
|  | | | | | | |

Table S3. Correlations of the 4 topic items of Benefits of Mixed Reality in the surgical field.

|  | | | COMMUNICATION | EFFICIENCY | SECURITY | PRECISION |
| --- | --- | --- | --- | --- | --- | --- |
| Kendall's tau_b | COMMUNICATION | Correlation Coefficient | -- |  |  |  |
|  |  | Sig. (2-tailed) | . |  |  |  |
|  |  | N | 20 |  |  |  |
|  | EFFICIENCY | Correlation Coefficient | -,435^a^ | -- |  |  |
|  |  | Sig. (2-tailed) | ,039 | . |  |  |
|  |  | N | 20 | 20 |  |  |
|  | SECURITY | Correlation Coefficient | ,202 | -,740^b^ | -- |  |
|  |  | Sig. (2-tailed) | ,347 | <,001 | . |  |
|  |  | N | 20 | 20 | 20 |  |
|  | PRECISION | Correlation Coefficient | -,285 | ,080 | -,410 | -- |
|  |  | Sig. (2-tailed) | ,192 | ,702 | ,054 | . |
|  |  | N | 20 | 20 | 20 | 20 |
| ^a^Correlation is significant at the 0.05 level (2-tailed). | | | | | | |
| ^b^Correlation is significant at the 0.01 level (2-tailed). | | | | | | |

Table S4. Correlations of the 5 topic items of Limitations in the implementation of Mixed Reality in the surgical area.

|  | | | DATA_ PROTECTION | REGULATORY | COSTS | HUMANS | TECHONOLOGICAL |
| --- | --- | --- | --- | --- | --- | --- | --- |
| Kendall's tau_b | DATA_PROTECTION | Correlation Coefficient | -- |  |  |  |  |
|  |  | Sig. (2-tailed) | . |  |  |  |  |
|  |  | N | 20 |  |  |  |  |
|  | REGULATORY | Correlation Coefficient | ,249 | -- |  |  |  |
|  |  | Sig. (2-tailed) | ,240 | . |  |  |  |
|  |  | N | 20 | 20 |  |  |  |
|  | COSTS | Correlation Coefficient | -,476^a^ | -,246 | -- |  |  |
|  |  | Sig. (2-tailed) | ,027 | ,241 | . |  |  |
|  |  | N | 20 | 20 | 20 |  |  |
|  | HUMANS | Correlation Coefficient | -,166 | ,081 | -,098 | -- |  |
|  |  | Sig. (2-tailed) | ,442 | ,701 | ,645 | . |  |
|  |  | N | 20 | 20 | 20 | 20 |  |
|  | TECHONOLOGICAL | Correlation Coefficient | ,048 | -,457^a^ | -,416^a^ | -,214 | -- |
|  |  | Sig. (2-tailed) | ,816 | ,024 | ,041 | ,296 | . |
|  |  | N | 20 | 20 | 20 | 20 | 20 |
| ^a^Correlation is significant at the 0.05 level (2-tailed). | | | | | | | |

Table S5. Correlations of the 5 topic items of Ethical considerations in the use of Mixed Reality in the surgical area.

|  | | | CONSENT | SECURITY | RESPONSIBILITY | PRIVACY |
| --- | --- | --- | --- | --- | --- | --- |
| Kendall's tau_b | CONSENT | Correlation Coefficient | -- |  |  |  |
|  |  | Sig. (2-tailed) | . |  |  |  |
|  |  | N | 20 |  |  |  |
|  | SECURITY | Correlation Coefficient | -,260 | -- |  |  |
|  |  | Sig. (2-tailed) | ,207 | . |  |  |
|  |  | N | 20 | 20 |  |  |
|  | RESPONSIBILITY | Correlation Coefficient | -,228 | -,416^a^ | -- |  |
|  |  | Sig. (2-tailed) | ,260 | ,044 | . |  |
|  |  | N | 20 | 20 | 20 |  |
|  | PRIVACY | Correlation Coefficient | -,188 | -,132 | -,279 | -- |
|  |  | Sig. (2-tailed) | ,350 | ,519 | ,167 | . |
|  |  | N | 20 | 20 | 20 | 20 |
| ^a^Correlation is significant at the 0.05 level (2-tailed). | | | | | | |
